# An Enhanced Monomeric Blue Fluorescent Protein with the High Chemical Stability of the Chromophore

**DOI:** 10.1371/journal.pone.0028674

**Published:** 2011-12-08

**Authors:** Oksana M. Subach, Paula J. Cranfill, Michael W. Davidson, Vladislav V. Verkhusha

**Affiliations:** 1 Department of Anatomy and Structural Biology, and Gruss-Lipper Biophotonics Center, Albert Einstein College of Medicine, New York, New York, United States of America; 2 National High Magnetic Field Laboratory and Department of Biological Science, The Florida State University, Tallahassee, Florida, United States of America; Stanford, United States of America

## Abstract

Commonly used monomeric blue fluorescent proteins suffer from moderate brightness. The brightest of them, mTagBFP, has a notably low chemical stability over time. Prolonged incubation of mTagBFP leads to its transition from a blue fluorescent state with absorbance at 401 nm to a non-fluorescent state with absorbance at 330 nm. Here, we have determined the chemical structure of the degraded product of the blue mTagBFP-like chromophore. On the basis of mTagBFP we have developed an improved variant, named mTagBFP2. mTagBFP2 exhibits 2-fold greater chemical stability and substantially higher brightness in live cells than mTagBFP. mTagBFP2 is also 1.2-fold and 1.7-fold more photostable than mTagBFP in widefield and confocal microscopy setups, respectively. mTagBFP2 maintains all other beneficial properties of the parental mTagBFP including the high pH stability and fast chromophore formation. The enhanced photostability and chromophore chemical stability of mTagBFP2 make it a superior protein tag. mTagBFP2 performs well in the numerous protein fusions and surpasses mTagBFP as a donor in Förster resonance energy transfer with several green fluorescent protein acceptors.

## Introduction

A number of fluorescent proteins (FPs) with different chromophores have been developed [1], [2]. Ultramarine, blue and cyan variants of a green fluorescent protein (GFP) contain the substitutions of tyrosine in the chromophore tripeptide with phenylalanine, histidine and tryptophan [3]–[5]. Blue color variants of various red fluorescent proteins (RFPs) with the Tyr-containing chromophore have been obtained [6]. The chemical structure of the brightest of these blue fluorescent proteins (BFPs), mTagBFP, has been determined [7]. mTagBFP has a novel type of chromophore, which contains the N-acylimine but does not have the C^α^-C^β^ double bond in the Tyr64 side chain. Therefore, the Tyr64 side chain is not part of the blue chromophore in mTagBFP.

A β-barrel protein structure in FPs creates a semi-rigid environment around the chromophore from where bulk solvent molecules are excluded, and a conformational flexibility of the chromophore is low [8]. Study of the unfolding of the GFP variant, trGFPuv [8], in the presence of the denaturant showed that energetic barriers are high and unfolding rates are very low in comparison to most small monomeric proteins. Therefore, FPs are biochemically very stable. However, stability of FPs inside mammalian cells is affected by proteolysis of the ubiquitin-proteasome system and autophagy [9]. The half-life of GFP is about 26 h in mammalian cells [10].

A crystal structure of a blue-to-red fluorescent timer, called Fast-FT, and its mutagenesis showed that in addition to the transformation into a red species over time, the blue Fast-FT chromophore underwent an unusual transformation into a degraded moiety, which absorbed at 320 nm [11]. An mTagBFP crystal structure demonstrated a slightly diffused electron density map of Leu63 that might also be caused by a minor degradation of its chromophore [7]. Maturation of DsRed.T7 protein indicated the formation from the blue chromophore both the non-absorbing species and the red chromophore species [12]. Overall, this raises a question about the stability of the blue mTagBFP-like chromophore and structure of its degraded (hydrolyzed) product.

All FPs undergo photobleaching upon prolonged light irradiation [13]. Until recently, use of BFPs has been limited by its rapid photobleaching. Several BFPs with improved photostability, such as Azurite [14], EBFP2 [15] and mTagBFP [6] have emerged over recent years. However, photochemical stability of the brightest mTagBFP was 1.6-fold lower than that for EBFP2 [6].

In this study, we revealed a chemical structure of the mTagBFP degradation product and developed a new BFP variant, named mTagBFP2, which exhibited higher chemical stability and better photochemical characteristics *in vitro* and in mammalian cells.

## Results and Discussion

### Development of mTagBFP2

**Figure 1:**
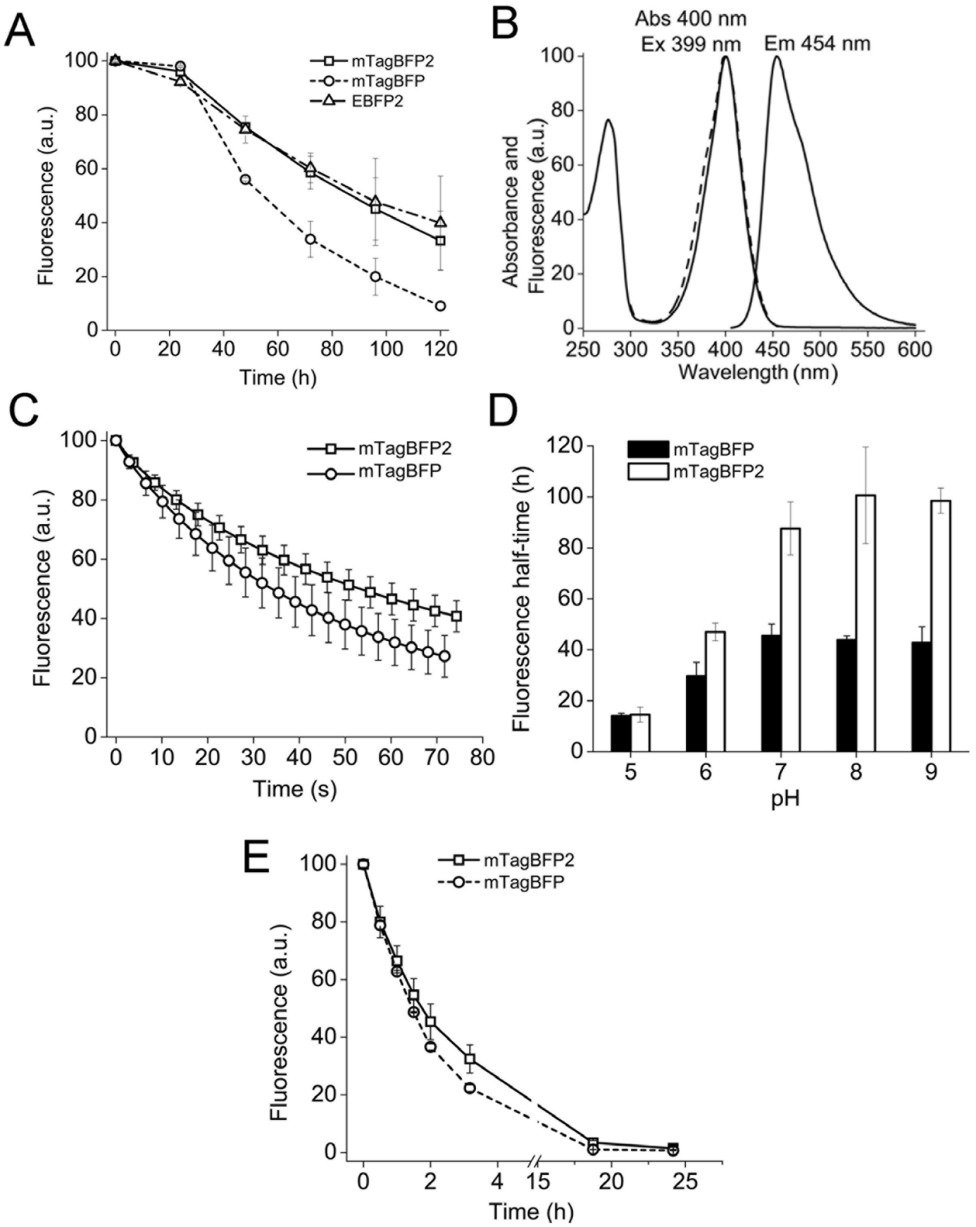
Photochemical and biochemical properties of the purified mTagBFP2 protein. (A) Time dependence of fluorescence for mTagBFP2, mTagBFP and EBFP2 in PBS, pH 7.4 at 37°C. (B) Excitation (dashed line), absorbance and emission (solid lines) spectra of mTagBFP2. (C) Photobleaching curves for purified mTagBFP2 (squares) and mTagBFP (circles) under epifluorescence illumination using metal halide arc lamp. According to the Student's t-test [21] a difference between the photobleaching curves is statistically significant. (D) Dependence of fluorescence half-life times on pH for mTagBFP2 and mTagBFP at 37°C. (E) Time dependence of fluorescence for mTagBFP2 and mTagBFP denatured in 6 M guanidinium hydrochloride at 25°C. Error bars, s.d. (n = 3 (A, D, E); and n = 10 (C)).

First, we tested stability of mTagBFP in a phosphate buffer saline (PBS) (Figure 1A). The control EBFP2 (fluorescence half-life time was 99±30 h) was more stable than mTagBFP (fluorescence half-life time was 54±4 h). This prompted us to increase chemical stability of mTagBFP. Previously, we observed that replacement of Tyr in the chromophore tripeptide with different aliphatic amino acid residues did not influence spectral properties of the mTagBFP chromophore [7]. However, we noted that blue fluorescence decreased after storage of these mutants. This instability possibly occurred because of an increased conformational flexibility of the blue chromophore with aliphatic amino acid residues in the chromophore tripeptide. With a rationale that the flexibility of the Tyr side chain may influence the chemical stability of the blue chromophore, we subjected three amino acid residues proximal to Tyr64 such as Phe144, Asn159, and Ile174 to mutagenesis [7]. Chemical stabilities of the brightest and most photostable mTagBFP mutants were checked. The best mTagBFP mutant, named mTagBFP2, contained a single I174A substitution and had excitation/emission maxima at 399/454 nm, which are similar to the parental protein (Figure 1B and Table 1). The fluorescence half-life time for mTagBFP2 was 88±14 h, which was substantially greater than that for mTagBFP (Figure 1A).

**Table 1:** Properties of purified mTagBFP2 in comparison with other monomeric blue fluorescent proteins.

Protein	Excitation maximum, nm	Emission maximum, nm	Extinction coefficient, M^−1^ cm^−1^	Quantum yield	Brightness relative to mTagBFP	Fluorescence lifetime, ns	Effective p*K*_a_	Photobleaching t_1/2_, sec
**Azurite**	383	447	26,200^a^	0.55^a^	0.55	3.4±0.2	5.0	33
**EBFP2**	383	448	32,000^b^	0.56^b^	0.69	3.0±0.2	4.5	55^b^
**mTagBFP**	399	456	41,400±200^c^	0.63±0.03	1.00±0.05	2.6±0.1	2.7±0.2	34±8
**mTagBFP2**	399	454	50,600±800	0.64±0.03	1.22±0.06	2.6±0.1	2.7±0.2	53±9

a Data from [14].

b Data from [15].

c Errors, s.d.

mTagBFP2 was brighter than parental mTagBFP due to its higher extinction coefficient (Table 1). We measured photostabilities of purified mTagBFP and mTagBFP2, and compared them using the standard procedure [13]. mTagBFP2 was 1.5-fold more photostable than mTagBFP under arc lamp illumination (Figure 1C, Table 1). The fluorescence lifetimes, effective p*K*a, and maturation half-times were approximately the same for both BFPs (Table 1, Figure S1).

The Ile174Ala mutation in mTagBFP2 possibly provides a free space near Tyr64, which can be filled up with a water molecule that results in changes of a hydrogen network in the chromophore environment and/or in stabilization of the Tyr64 side chain by the additional hydrogen bond. Another possibility is that the free space can be favorable for conformational changes in the chromophore immediate environment. Both scenarios can favor the stability of the chromophore in mTagBFP2.

### Properties of purified mTagBFP2

We characterized the chemical stability of mTagBFP and mTagBFP2 at different pH values and in a denaturating agent. First, we measured the half-life times of purified mTagBFP and mTagBFP2 at different pH values. At pH 5, the stabilities of both BFPs were similar (Figure 1D). An increase of pH resulted in the increase of the difference in stabilities for the proteins. At pH 7–9, the stability of mTagBFP2 was about 2-fold greater than that of mTagBFP. Since our data on chemical stabilities of mTagBFP2 and mTagBFP were based on measurement of the blue fluorescence, the measured stabilities can be attributed to a chemical stability of the mTagBFP2 and mTagBFP chromophores.

Guanidinium hydrochloride and elevated temperatures are commonly used to study the stability of FPs *in vitro* [8], [16]. Unfolding of purified mTagBFP and mTagBFP2 in 6 M guanidinium hydrochloride showed that the half-life time of mTagBFP2 was 1.74±0.23 h, which was slightly greater than that of mTagBFP (1.43±0.01 h) (Figure 1E). This suggests that the mTagBFP2 polypeptide is more stable. Altogether our data showed that mTagBFP2 was more stable than mTagBFP, and that the higher stability of the mTagBFP2 chromophore correlated with the increased stability of its polypeptide.

**Figure 2:**
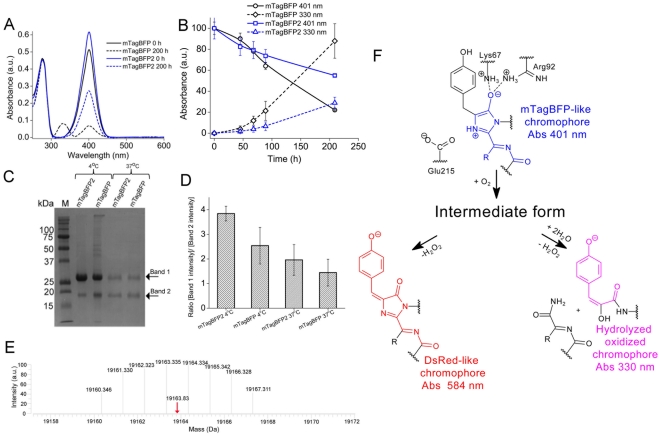
Autocatalytic degradation of the TagBFP-like chromophore. (A) Absorbance spectra of mTagBFP2 (blue) and mTagBFP (black) before and after incubation in 100 mM NaH_2_PO_4_, 300 mM NaCl, pH 8.0 at 37°C for 200 h. (B) Time dependence of absorbance for mTagBFP2 (blue) and mTagBFP (black) at 330 nm (dashed lines) and 401 nm (solid lines). Purified proteins were incubated in 100 mM NaH_2_PO_4_, 300 mM NaCl, pH 9.0 at 37°C. (C) SDS-PAGE analysis of mTagBFP2 and mTagBFP after incubation at 4°C (lanes 2 and 3) or at 37°C (lanes 4 and 5) for 770 h. M: molecular weight protein markers. Bands 1 and 2 show the total polypeptide chain and the polypeptide chain after cleavage inside the chromophore, respectively. (D) The ratio of the band 1 intensity to the band 2 intensity for SDS-PAGE analysis presented in Figure 2C. (E) Deconvoluted FT-ICR mass spectrum: isotopic distribution of the region corresponding to the band 2 (C). The deconvoluted average mass (19163.83 Da) is indicated by the red arrow. (F) Chemical scheme of hydrolysis of the mTagBFP-like chromophore. Error bars, s.d. (n = 2 (B); and n = 3 (D)).

A prolonged incubation of both BFPs at 37°C led to the transition from the fluorescent state with the absorbance maximum at 401 nm to a non-fluorescent state with the absorbance maximum at 330 nm (Figure 2A). mTagBFP converted to the non-fluorescent form faster than mTagBFP2 (Figure 2B). However, a transition of the mTagBFP-like chromophore to the DsRed-like chromophore was not observed for both BFPs (Figure 2A). The prolonged incubation of mTagBFP and mTagBFP2 resulted in an accumulation of their cleaved product with a molecular weight of ∼19 kDa (Figure 2C). This molecular weight corresponded to the mTagBFP or mTagBFP2 polypeptides that are cleaved around the chromophore. The 19 kDa product was more prominent in the case of mTagBFP (Figure 2D).

Earlier studies of the blue-to-red Fast-FT suggested that its blue chromophore converted into two forms, the red chromophore and the degraded chromophore moiety with an absorbance at 320 nm [11]. Our data suggest that after degradation the mTagBFP and mTagBFP2 chromophores convert into the latter moiety. To determine the chemical structure of the degraded product, we subjected mTagBFP to Fourier-transform ion cyclotron resonance mass spectrometry (FT-ICR MS) after prolonged incubation at 4°C (lane 3 in Figure 2C). A deconvolution of the FT-ICR mass spectrum revealed a mass of 19163.83 Da (Figure 2E). This mass can correspond to the chemical structure of the hydrolyzed form (Figure 2F), which has the theoretical mass of 19163.65 Da. The discovered structure is similar to that proposed earlier for Fast-FT [11] except for an amino group at the C^α^ position of Tyr64 that is replaced by a hydroxyl group. Such degraded product may form due to an oxidation of the Tyr side chain and hydrolysis of the imidazole residue in the chromophore. According to the crystal structure of mTagBFP [7], the nitrogen atom of the imidazole residue forms a hydrogen bond with a water molecule, which is hydrogen bonded to Glu215. This amino acid possibly catalyzes the oxidation and hydrolysis of the mTagBFP-like chromophore.

We suggest that a common intermediate is formed from the blue mTagBFP-like chromophore (Figure 2F). The intermediate can either transform into the red DsRed-like chromophore after oxidation of the C^β^ atom of Tyr64 or into the hydrolyzed product with the absorbance at 330 nm after oxidation of the C^β^ atom of Tyr64 and hydrolysis of the imidazole ring. The hydrolyzed product can be in equilibrium with the compound containing a carbonyl group instead of an enol group (Figure S2A). The second compound, which is formed together with the 330 nm absorbing hydrolyzed product (Figure 2F), can be further hydrolyzed at the N-acylimine (Figure S2B). DsRed-like red chromophore is not formed in mTagBFP2 possibly because a bulky side chain of Phe143 pushes the chromophore's hydroxyphenyl group out of the plane of the imidazole-5-ol ring. This prevents formation of the C^α^-C^β^ double bond in the Tyr64 side chain, which is coplanar with the tyrosine phenolate and the imidazole-5-ol ring [7].

### Behavior of mTagBFP2 in live cells

**Figure 3:**
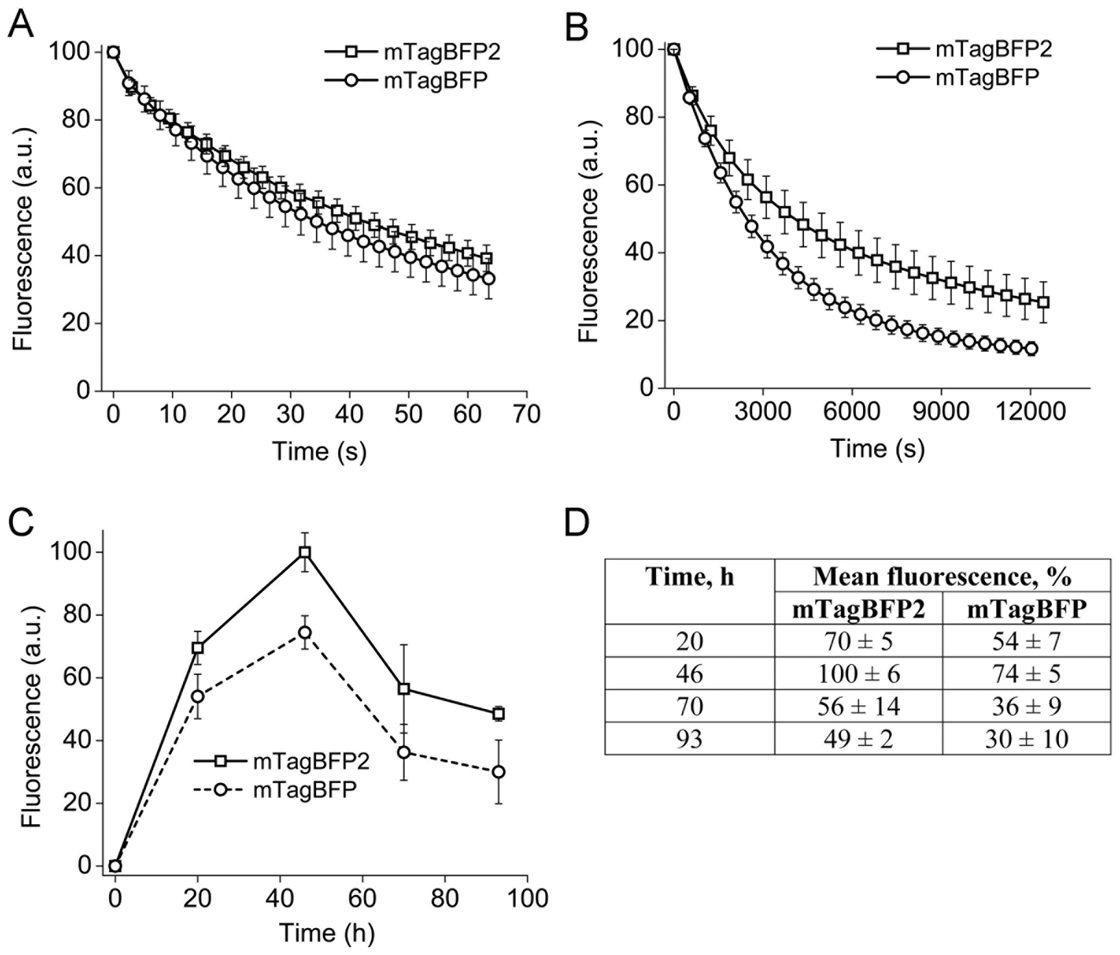
Properties of mTagBFP2 in live mammalian cells. Photobleaching curves for mTagBFP2 (squares) and mTagBFP (circles) expressed in HeLa cells under metal halide arc illumination (A) or 405 nm laser scanning confocal microscope illumination (B). Data represent an average of 32–65 cells per each protein. According to the Student's t-test [21] a difference between the photobleaching curves is statistically significant. (C) Mean fluorescence brightness of HeLa cells expressing mTagBFP2 (solid line) or mTagBFP (dashed line). (D) Mean fluorescence of mTagBFP2- and mTagBFP-expressing HeLa cells corresponding to panel (C). Error bars, s.d. (n = 65 (A); n = 32 (B); and n = 3 (C)).

Widefield (Figure 3A) and laser scanning confocal (Figure 3B) photobleaching experiments in live mammalian cells were conducted with N-terminal fusions of mTagBFP and mTagBFP2 to human histone H2B. The photostability of mTagBFP2 (43±5 s) was higher than that of mTagBFP (35±6 s) in the wide-field microscopy experiment. In confocal microscopy the photostability of mTagBFP2 was more substantial: 4152±871 s for mTagBFP2 versus 2477±405 s for mTagBFP.

To compare brightness of mTagBFP and mTagBFP2 in live cells, both proteins were expressed in the cytosol of live HeLa cells, and the mean fluorescence of the cells was compared using flow cytometry (Figure 3C–D). The mTagBFP2-expressing cells were notably brighter than the mTagBFP cells, and the maximal brightness was achieved at 46 h after the cell transfection with BFPs. Figure 3D shows that the ratio of the mean fluorescence of mTagBFP2 to the mean fluorescence of mTagBFP was increased with time from 1.3 at 20 h after transfection to 1.6 at 70–93 h after transfection. This increase is consistent with the improved chemical stability of mTagBFP2.

**Figure 4:**
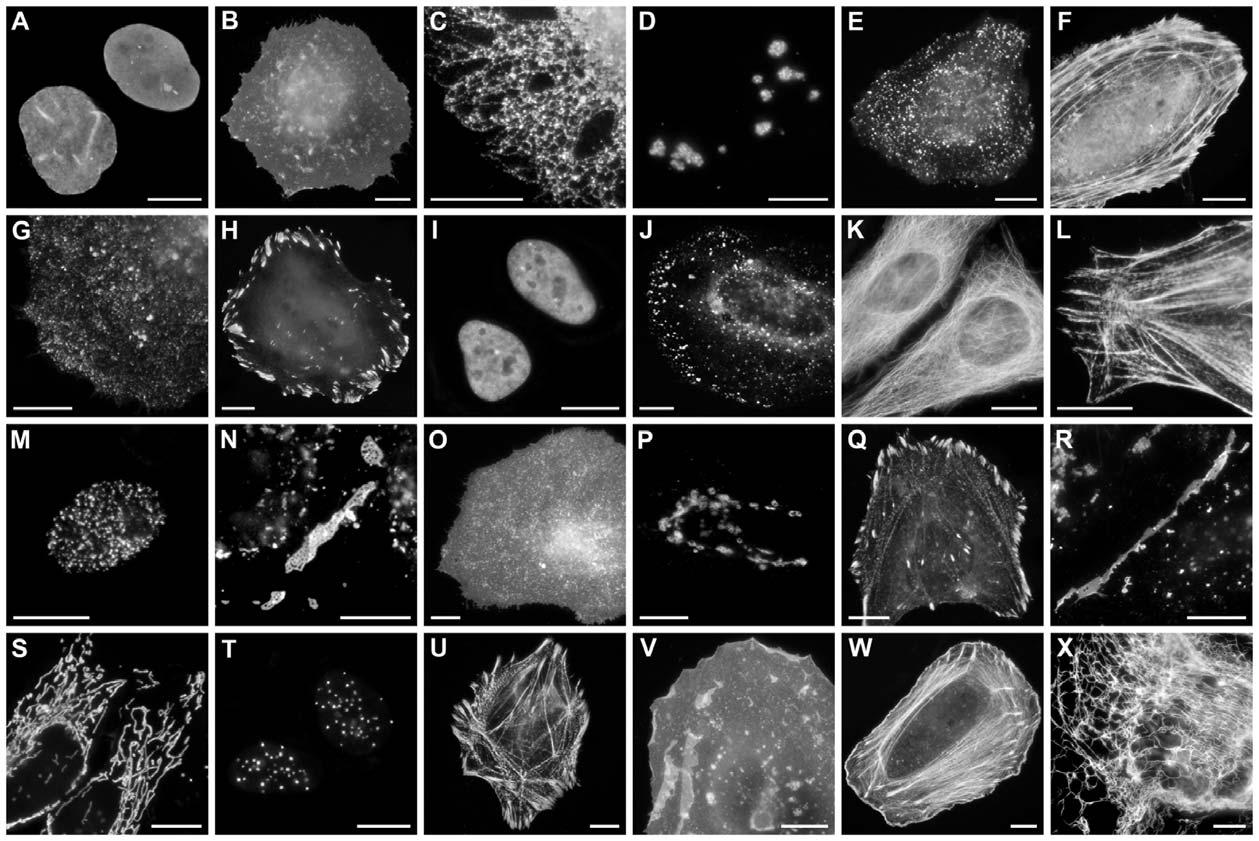
Fluorescence imaging of mTagBFP2 fusion constructs. (A–M) *C-terminal fusion constructs*. For each fusion protein, the linker amino acid length is indicated after the name of the targeted organelle or fusion protein. (A) mTagBFP2-lamin B1-10; (B) mTagBFP2-CAAX-farnesyl-5; (C) mTagBFP2-endoplasmic reticulum-5 (calreticulin and KDEL); (D) mTagBFP2-fibrillarin-7; (E) mTagBFP2-light chain clathrin-15; (F) mTagBFP2-β-actin-7; (G) mTagBFP2-caveolin 1-10; (H) mTagBFP2-vinculin-22; (I) mTagBFP2-CAF1-10 (chromatin assembly factor); (J) mTagBFP2-Rab5a-7; (K) mTagBFP2-α-tubulin-18; (L) mTagBFP2-myosin-IIA-18; (M) mTagBFP2-PCNA-19. (N–X) *N-terminal fusion constructs*. (N) Cx26-mTagBFP2-7; (O) TfR-mTagBFP2-20 (transferrin receptor); (P) Golgi-mTagBFP2-7; (Q) zyxin-mTagBFP2-6;(R) VE cadherin-mTagBFP2-10; (S) mitochondria-mTagBFP2-7; (T) CENPB-mTagBFP2-22; (U) α-actinin-mTagBFP2-19; (V) c-src-mTagBFP2-7; (W) Lifeact-mTagBFP2-7; (X) vimentin-mTagBFP2-7. The cell line used for expressing mTagBFP2 fusion vectors was opossum kidney cortex proximal tubule epithelial cells (ATCC CRL-1840) in panel X, and human cervical adenocarcinoma cells (HeLa; ATCC CCL-2) in the remaining panels. The scale bar in each panel equals 10 µm.

Because the I174A substitution in mTagBFP2 was interior to the protein β-can fold, we anticipated that it should not influence the performance of mTagBFP2 as a protein fusion tag. Indeed, mTagBFP2 did not interfere with the localization of 24 various fusion constructs (Figure 4).

**Figure 5:**
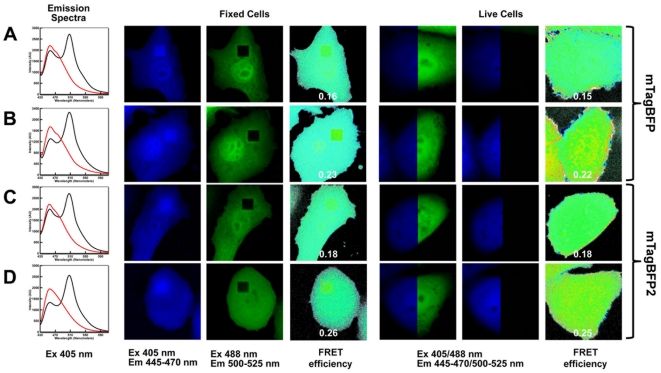
Behavior of mTagBFP and mTagBFP2 as FRET donors. (A) mTagBFP-mEGFP; (B) mTagBFP-mEmerald; (C) mTagBFP2-mEGFP; (D) mTagBFP2-mEmerald. Left column: emission spectra of FRET pairs with excitation at 405 nm before (black lines) and after (red lines) acceptor photobleaching. Columns 2 and 3: fixed cells in blue (Ex. 405 nm, Em. 445–470 nm) and green (Ex. 488 nm, Em. 500–535 nm) fluorescence channels after acceptor photobleaching in the square region (black square in the green channel). Column 4: FRET efficiency in fixed cells. Columns 5–6: live cells in blue/green channel before (column 5) and after (column 6) acceptor photobleaching are shown. Column 7: FRET efficiency in live cells. Colored scale bars (columns 4 and 7) show relative FRET efficiencies. See also Table 2.

To characterize mTagBFP2 as a FRET donor, we compared it with mTagBFP in the FRET pairs with mEGFP and mEmerald acceptors in fixed and live cells (Figure 5). For FRET measurements we used the acceptor photobleaching method [17]. In spite of rather similar quantum yields observed for the freshly purified BFPs the mTagBFP2 protein provided 1.13–1.2-fold better FRET efficiency than mTagBFP (Table 2). Taking into account the lower chemical stability of mTagBFP we measured a dependence of efficient quantum yields of the purified BFPs with time. An efficient quantum yield is the ensemble parameter that averages the quantum yields of all types of species present in the sample. The efficient quantum yield of the mTagBFP2 sample did not change over time. However, the efficient quantum yield of the mTagBFP sample started decreasing after 45 h of incubation (Figure S3) suggesting that its conversion into the similarly absorbing intermediate form (Figure 2F) occurs substantially faster. In other words, the mTagBFP sample contained a notable amount of another species already after 45 h. A chromophore of the intermediate form may be less planar or more mobile, resulting in the increase of the radiationless transition from the first excited state to the ground state and, subsequently, in the lower quantum yield of these intermediate species. Therefore, the better FRET efficiency in FRET pairs with mTagBFP2 donor can be explained by the substantially higher chemical stability of its chromophore with time. The best FRET pair tested was mTagBFP2-mEmerald. The FRET efficiency for the widely used cyan-yellow construct, mCerulean-mVenus, was the same [18].

**Table 2:** Analysis of FRET efficiencies in live and fixed HeLa cells.

FRET Pair	Average FRET Efficiency			
	Live Cell		Fixed Cell	
	Average	Standard Deviation	Average	Standard Deviation
mTagBFP-mEGFP	0.15	0.02	0.16	0.01
mTagBFP-mEmerald	0.22	0.02	0.23	0.01
mTagBFP2-mEGFP	0.18	0.02	0.18	0.01
mTagBFP2-mEmerald	0.25	0.01	0.26	0.02

In conclusion, the chemical structure for the degraded product of the mTagBFP-type chromophore was revealed. The enhanced brightness, photostability and chromophore stability of the mTagBFP2 protein make it a superior probe for fluorescence microscopy. Moreover, live cell imaging showed that all tested mTagBFP2 fusions with cellular proteins properly localize in a cell. Lastly, mTagBFP2 is proved to be an excellent FRET donor for mEmerald.

## Materials and Methods

### Cloning, expression, and protein purification

For bacterial expression of mTagBFP or mTagBFP2, a pBAD/HisB vector (Invitrogen) was modified by shortening the N-terminal His_6_-tag to the MGSHHHHHHGRS- amino acids. The PCR-amplified *Bgl*II/*Eco*RI fragment encoding mTagBFP or mTagBFP2 was cloned into the modified pBAD/HisB vector and expressed in LMG194 host (Invitrogen). The bacterial culture in RM minimal medium supplemented with 0.005% arabinose was grown overnight at 37°C. The culture was centrifuged at 5,000 rpm at 4°C for 15 min. The cell pellet was resuspended in PBS, 300 mM NaCl, pH 7.4 and lysed by sonication on ice. The recombinant protein was purified using Ni-NTA resin (Qiagen) followed by dialysis for 3 h against PBS.

### Mutagenesis and screening of libraries

For simultaneous mutagenesis at several positions of the mTagBFP gene the overlap-extension approach has been applied [19]. After mutagenesis a mixture of the mutants was electroporated into LMG194 host cells (Invitrogen)

Protein expression in the library was induced overnight at 37°C with 0.002% arabinose. Next morning, expressing bacteria were washed with PBS and then diluted with PBS for FACS sorting to optical density of 0.02 at 600 nm. MoFlo cell sorter (Dako) equipped with standard argon, krypton and argon-krypton mixed-gas lasers was used. To select the most photostable clones the library was illuminated before FACS using LED array at 405 nm for 15 min. About 10 sizes of the library were sorted on the FACS with 407 nm of krypton excitation line and 450/65 nm emission filter. The collected bright blue bacterial cells were rescued in rich SOC medium at 37°C for one hour, and then plated on Petri dishes with 0.02% arabinose. The next day, the dishes were analyzed with Leica MZ16F fluorescence stereomicroscope using a custom blue filter set (390/40 nm exciter, 460/40 nm emitter) from Chroma. To select the most photostable colonies, dishes were illuminated with 405 nm LEDs (80 mW/cm^2^) for 15 min, and images were acquired before and after illumination. For further analysis, 20 to 50 brightest and photostable clones were selected and analyzed using Olympus IX81 inverted microscope equipped with a 200 W metal-halide lamp (Prior), 100×1.4 NA oil objective lens (UPlanSApo, Olympus), and 390/40 nm excitation and 460/40 nm emission filters. At this stage photobleaching of the blue bacterial clones was checked. The best clones were applied for sequencing.

### Characterization of purified proteins

Absorbance spectra were recorded on a U-3010 spectrophotometer (Hitachi). The excitation and emission spectra were measured using a FluoroMax-3 spectrofluorometer (Jobin Yvon). For measurements the protein samples in PBS were used. Quantum yield was measured using mTagBFP (quantum yield is 0.63 [6]) as the reference standard. Protein concentrations used in the calculation of extinction coefficients were determined by the BCA assay (Pierce). Brightness of BFPs was calculated as the product of the Extinction coefficient (EC) and the Quantum Yield (QY). For calculation of the errors for Brightness (EC*QY) we used the following formula: EC*(s.d. of QY)+QY*(s.d. of EC).

Equilibrium pH titrations were performed using a series of buffers (100 mM NaOAc, 300 mM NaCl for pH 2.5–5.0, and 100 mM NaH_2_PO_4_, 300 mM NaCl for pH 4.5–9.0).

Photobleaching kinetics was measured using purified proteins in PBS at 1 mg/ml in aqueous drops in oil using Olympus IX81 inverted microscope equipped with the 200 W metal halide arc lamp, 100×1.4 NA oil immersion objective lens, 390/40 nm excitation and 460/40 nm emission filters. The microscope was operated with SlideBook 4.2 software (Intelligent Imaging Innovations). Light power densities were measured at a rear focal plane of the objective lens. The data were normalized to a spectral output of the lamp, transmission profiles of the filters and dichroic mirror, and absorbance spectra of the respective proteins. To calculate a photobleaching half-time, the experimental dependence of fluorescence on time was fitted with a mono-exponential function, and a time corresponding to 50% of maximal fluorescence was found using the fitting function.

To study protein maturation, LMG194 bacteria transformed with the mTagBFP or mTagBFP2 genes were grown in an RM medium supplemented with ampicillin at 37°C overnight. The next morning, bacterial cells were diluted to optical density 1.0 at 600 nm, and 0.2% arabinose was added. Upon induction of protein expression, bacterial cultures were grown at 37°C in 50 ml tubes filled to the brim and tightly sealed to restrict oxygen supply. After 1 hour, the cultures were centrifuged in the same tightly closed tubes. After opening the tubes, the proteins were purified using the Ni-NTA resin within 30 min, with all procedures and buffers at or below 4°C. Protein maturation occurred in PBS at 37°C. Blue fluorescence signal of the proteins was monitored using the FluoroMax-3 spectrofluorometer.

To study the chemical stability of mTagBFP and mTagBFP2 at 37°C, purified proteins at concentrations of 0.5 mg/ml for absorbance and 0.05 mg/ml for fluorescence measurements in PBS or 100 mM NaH_2_PO_4_, 300 mM NaCl pH 5.0–9.0 were used. For denaturation measurements, mTagBFP or mTagBFP2 at concentrations 0.05 mg/ml for fluorescence measurements were incubated in 6 M guanidinium hydrochloride, pH 7.4 at 25°C. To calculate half-life times for mTagBFP2 and mTagBFP, the experimental dependences of fluorescence on time were fitted with mono-exponential functions, and times corresponding to 50% of maximal fluorescence were determined using the fitting functions.

### Mammalian plasmids and cell culture

mTagBFP2 fluorescent protein expression vectors were constructed using -C1 and -N1 (Clontech-style) cloning vectors. The mTagBFP2 cDNA was amplified with a 5′ primer encoding an *Age*I site and a 3′ primer encoding either a *Bsp*EI (-C1) or *Not*I (-N1) site for C-terminal and N-terminal fusions (with regards to the FP), respectively. Purified and digested PCR products were ligated into similarly digested pEGFP-C1 and pEGFP-N1 cloning vector backbones. To generate targeting fusion vectors, the appropriate cloning vector and a previously assembled EGFP fusion vector were digested, either sequentially or doubly, with the appropriate enzymes and ligated together after gel purification.

Thus, to prepare mTagBFP2 C-terminal fusions (number of linker amino acids in parenthesis), the following digests were performed: human lamin B1 (10), *Nhe*I and *Bgl*II (lamin B1 cDNA source: George Patterson, NIH; NM_005573.2); 20 amino acid farnesylation signal from c-Ha-Ras (CAAX; 5), *Age*I and *Bsp*EI (c-Ha-Ras cDNA source: Clontech, Mountain View, CA; NM_001130442.1); endoplasmic reticulum (5), *Age*I and *Bsp*EI (calreticulin cDNA source: George Patterson, NIH; NM_004343.3); fibrillarin (7), *Age*I and *Bsp*EI (fibrillarin cDNA source: Evrogen, Moscow, Russia; NM_001436.3); human light chain clathrin (15), *Nhe*I and *Bgl*II (clathrin cDNA source: George Patterson, NIH; NM_001834.2); β-actin (7), *Nhe*I and *Bgl*II (human β-actin cDNA source: Clontech, Mountain View, CA; NM_001101.3); caveolin 1 (10), *Nhe*I and *Bgl*II (human caveolin 1 cDNA source: Origene, Rockville, MD; NM_001753); vinculin (22) *Age*I and *Eco*RI (human vinculin source: Clare Waterman, NIH; NM_003373.3); CAF1 (10), *Age*I and *Bsp*EI (mouse chromatin assembly factor cDNA source: Akash Gunjan, FSU; NM_013733.3) Rab5a (7), *Nhe*I and *Bgl*II (canine Rab5a cDNA source: Vicki Allen, University of Manchester; NM_001003317.1); α-tubulin (18), *Nhe*I and *Bgl*II (human α-tubulin cDNA source: Clontech, Mountain View, CA; NM_006082); myosin IIA (18) *Nhe*I and *Bgl*II (human myosin heavy chain IIA cDNA source: DNA2.0, Menlo Park, CA; AJ312390.1); PCNA (19), *Age*I and *Bsp*EI (proliferating cell nuclear antigen cDNA source: David Gilbert, FSU; NM_002592.2).

To prepare mTagBFP2 N-terminal fusions (number of linker amino acids in parenthesis), the following digests were performed: β-2 connexin-26 (7), *Bam*HI and *Not*I (rat Cx26 cDNA source: Matthias Falk, Lehigh U; NM_001004099.1); TfR (20), *Bam*HI and *Not*I (transferrin receptor cDNA source: George Patterson, NIH; NM_NM_003234); Golgi complex (7), *Bam*HI and *Not*I (human β-galactosamide α-2,6-sialyltransferase 1cDNA source: Jennifer Lippincott-Schwartz, NIH; NM_173216.2); zyxin (6), *Bam*HI and *Not*I (human zyxin cDNA source: Origene, Rockville, MD; NM_003461); vascular epithelial cadherin (10), *Age*I and *Not*I (human VE cadherin cDNA source: Origene, Rockville, MD; NM_001795.3); mitochondria (7), *Bam*HI and *Not*I (human mitochondrial targeting sequence, cytochrome c oxidase cDNA source: Clontech, Mountain View, CA; NM_004074.2); centromere protein B (22), *Bam*HI and *Not*I (human CENPB cDNA source: Alexey Khodjakov, Wadsworth Center, Albany, NY; NM_001810.5); α-actinin (19), *Bam*HI and *Eco*RI (human α-actinin cDNA source: Tom Keller, Florida State University, Tallahassee; NM_001130005.1); c-src sarcoma (7), *Bam*HI and *Eco*RI (chicken c-src cDNA source: Marilyn Resh, Sloan-Kettering, New York; XM_001232484.1); Lifeact (7), *Bam*HI and *Not*I (Lifeact cDNA source: IDT, Coralville, IA); vimentin (7), *Bam*HI and *Not*I (human vimentin cDNA source: Robert Goldman, Northwestern University; NM_003380.3).

All DNA for transfection was prepared using the Plasmid Maxi kit (QIAGEN, Valencia, CA). To ensure proper localization, mTagBFP2 fusion proteins were characterized by transfection in HeLa cells (CCL2 line; ATCC, Manassas, VA) using Effectene (QIAGEN) and 1 µg vector. Transfected cells were grown on coverslips in DMEM/F12, fixed after 48 hours, and mounted with Gelvatol. Epifluorescence images (Figure 4) were taken with a Nikon 80i microscope using widefield illumination and an Omega QMax Blue filter set to confirm proper localization.

### Characterization of proteins in mammalian cells

To prepare for FACS analysis, HeLa cells were transfected using Effectene (QIAGEN) and 400 ng of pmTagBFP-N1 and pmTagBFP2-N1 vectors in 6-well plates. 20, 46, 70 and 93 h after transfection cells were washed twice with PBS, treated with trypsin, suspended in PBS containing 4% of fetal bovine serum and analyzed by FACS.

To characterize photostability of mTagBFP2 and mTagBFP in HeLa cells, wide-field and laser scanning confocal microscopy photobleaching experiments were conducted with N-terminal fusions of mTagBFP and mTagBFP2 to human histone H2B to confine fluorescence to the nucleus in order to closely approximate the dimensions of aqueous droplets of purified FPs used in wide-field measurements [13]. Previously, this approach was applied for characterization of photostability of red fluorescent proteins in mammalian cells under laser scanning confocal microscope [20]. Photostability is time for photobleaching from an initial emission rate of 1,000 photons/s down to 500 photons/s [13]. First, time dependences of fluorescence were obtained. The data were normalized to a spectral output of the lamp, transmission profiles of the filters and dichroic mirror, and absorbance spectra of the respective proteins as previously described for wide-field microscopy experiment [13]. In the case of confocal microscopy experiment the data were normalized to the output power of the laser and the extinction coefficient at the laser wavelength [20]. To calculate a photobleaching half-time, the experimental dependence of fluorescence on time was fitted with a mono-exponential function, and a time corresponding to 50% of maximal fluorescence was found using the fitting function.

### FRET measurements

FRET constructs of mTagBFP/mTagBFP2 with mEGFP/mEmerald contained 10 amino acid linker -SGLRSPPVAT- between FPs, the same that was used in mCerulean:mVenus, mCerulean3:mVenus and mTurquoise:mVenus FRET constructs [18]. All acceptor photobleaching measurements were performed on an Olympus FV1000 confocal microscope with a UPLAPO 40× oil-immersion objective (1.0 NA). The 488 nm Argon laser line was used with a 405/488 dichroic mirror to excite and photobleach the mEGFP and mEmerald in FRET constructs. Emission was collected in one channel spanning 500–525 nm. For all of the FRET constructs, a 405 nm diode laser line was used with a 405/488 dichroic for excitation of the BFP with one emission channel spanning 445–470 nm. The detector gain was set to 500 V, the offset was set to 9 and the scan speed was set to 8.0 µs/pixel. Each experiment was performed with a pinhole size of 500 µm.

For FRET efficiency measurements in live cells, a full view image of the donor was acquired before and after acceptor photobleaching of the entire cell. The region of interest was drawn over the same part of the cell in each image, and the average intensities of these regions were calculated using the microscope's software. The following formula was used to calculate the FRET efficiency of each construct:
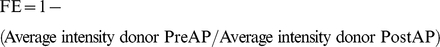



For FRET efficiency measurements in fixed cells, a full view image of the donor was acquired before and after acceptor photobleaching. The region of interest was drawn over a part of the cell and acceptor photobleached. The average intensities of these regions were calculated using the microscope's software and the above FE formula was again used to calculate FRET efficiency.

## Supporting Information

Figure S1 **The pH dependences of fluorescence and maturation curves for mTagBFP2 and mTagBFP.** (A) pH dependences. mTagBFP2 and mTagBFP were incubated at 25°C in a series of buffers (100 mM NaOAc, 300 mM NaCl for pH 2.5–5.0, and 100 mM NaH_2_PO_4_, 300 mM NaCl for pH 4.5–9.0) for 20 min followed by measurement of the blue fluorescence. The p*K*_a_ values of mTagBFP2 and mTagBFP are 2.7. (B) Maturation curves at 37°C in phosphate buffer saline (PBS), pH 7.4 are shown. The blue fluorescence was monitored using the FluoroMax-3 spectrofluorometer. The maturation half-time for mTagBFP2 and mTagBFP is 12 min and 13 min, respectively.
(TIF)

Figure S2 **Transformation of the degradation products of the mTagBFP chromophore.** (A) The suggested tautomeric forms of the mTagBFP hydrolyzed oxidized chromophore are shown. (B) The second polypeptide product of the mTagBFP degradation contains the N-acylimine, which can be further hydrolyzed to a pyruvamide derivative and a shorter polypeptide.
(TIF)

Figure S3 **The time dependence of the efficient quantum yields of mTagBFP2 and mTagBFP.** The mTagBFP2 and mTagBFP purified proteins were incubated at 37°C, and the quantum yields of both BFPs were measured at different time points. The efficient quantum yield is an ensemble parameter that averages the quantum yields of all types of species present in the sample. Here is the standard procedure for determining the quantum yields. To determine a quantum yield of FP, we plot the linear dependences of integrated fluorescence of FPs on absorbance at the excitation wavelength used to record the emission spectra. Then we determine the angles between the linear dependences and the x-axis. The tangent of this angle for FP provides a value of the quantum yield.
(TIF)
